# Municipal Support of Diabetes Management in Daycare, Kindergarten and School: A Qualitative Study of Differences, Challenges and Potentials

**DOI:** 10.3390/healthcare10081557

**Published:** 2022-08-17

**Authors:** Lise Bro Johansen, Anne Østergaard Nannsen, Mia Sørensen Iken, Mette Madsen, Kurt Kristensen, Kasper Ascanius Pilgaard, Anders Jørgen Schou, Stine Hangaard, Annette Korsholm Mouritsen, Anette Andersen, Dan Grabowski

**Affiliations:** 1 Steno Diabetes Center Copenhagen (SDCC), 2730 Herlev, Denmark; 2 Steno Diabetes Center Aarhus (SDCA), 8200 Aarhus, Denmark; 3 Department of Research, Danish Diabetes Association, 2600 Glostrup, Denmark; 4 Steno Diabetes Center Nordjylland (SDCN), 9000 Aalborg, Denmark; 5 Steno Diabetes Center Odense (SDCO), 5000 Odense, Denmark; 6 Steno Diabetes Center Sjælland (SDCS), 4000 Roskilde, Denmark

**Keywords:** diabetes, child, daycare, kindergarten, school, support, qualitative

## Abstract

Diabetes care during institutional hours is a major challenge affecting the whole family. The aim of this study was to highlight challenges and potentials regarding municipal support in relation to diabetes care of children in school, kindergarten, and daycare. The dataset consists of 80 semi-structured online interviews with 121 municipal employees from 74 (of 98) municipalities in Denmark. Data were analysed using qualitative content analysis. The analysis produced four main themes: (1) Institutional staff initially feel insecure about diabetes care responsibilities, (2) There is a high degree of parental involvement and responsibilities during institutional hours, (3) The roles of health employees vary, and (4) Fluctuating allocation of special needs assistants (SNAs) creates challenges. The findings of this nationwide qualitative study show that, even though Denmark guarantees, by law, the child’s right to support in diabetes self-care in school and childcare institutions, diabetes management in Denmark still needs to be improved, with a view to ensuring equal support for all children with diabetes.

## 1. Introduction

Children with type 1 diabetes who are attending daycare, kindergarten and school need special attention and support to manage their diabetes during the day. This entails checking blood glucose levels multiple times each day and potentially administering insulin or snack eating in the case of high or low glucose levels. If the blood glucose is not well regulated, it can lead to short-term complications, such as difficulties concentrating and restlessness, which lead to poor conditions for learning as well as long-term complications for, e.g., vision, kidney and nerve function [1,2].

As children in Denmark spend approximately 32 h a week in institutional settings, it is important that diabetes be well managed during institutional hours. Enhancing our understanding of how to mobilize support for children with diabetes in everyday contexts is an important area for research.

One Danish study elucidated associations between poor glycaemic control and poor psychological school-related wellbeing [3]. Avoidance of diabetes self-care during school hours is well recognized. Adolescence is a period in particular that is often characterized by reduced blood glucose testing and insulin omission related to young people’s desire to be ‘normal’ [4]. Moreover, research has suggested that children and adolescents with diabetes are more likely to experience bullying in school [5]. Research has also shown that children with diabetes want institutional staff to improve their knowledge about diabetes. Furthermore, children resent it when staff draw attention to them, because children do not want to feel singled out [6].

Diabetes care in institutional settings is a major challenge that affects the whole family. Research has shown that a high level of worry is experienced by parents while their child is at the institution. The parents find it difficult to balance the uncertainty of daily life with diabetes, as they feel they need to be available during institutional hours [7,8]. Parents also find it difficult to deal with time-consuming practices of seeking municipal funds, because the granted support varies across Danish municipalities [9]. Trusting and educating institutional staff constitute another major burden on parents [10]. One Swedish study demonstrated that parents of children with diabetes have significantly more burnout symptoms than parents of children without the disease [11]. Parents are less worried and more satisfied with diabetes self-care in school if there is a school staff member assigned to the child [8]. The presence of school nurses has been shown to affect parents’ and children’s sense of psychological and physical safety as well as to facilitate improved metabolic control [6,12,13].

There has been no qualitative research conducted regarding the views of municipal employees on paediatric diabetes support. Research on support and management of diabetes in schools has primarily focused on parents’ and the children’s perceptions and shown varying levels of support across institutions [3,5,6]. However, research looking at the perspective of municipality employees and their experience of municipal structures will provide insights into the municipal processes and how these affect institutional structures of support for children with diabetes nationally. Understanding ways to facilitate support of children with diabetes in institutional settings may improve children’s chances to receive equal support and their ability to manage diabetes during institutional hours, as well as further improving their learning environments. Hence, the overall objective of the present study was to highlight challenges and potentials regarding municipal support in relation to diabetes care for children in schools, kindergartens, and daycare in a Danish context.

## 2. Methods

A qualitative and descriptive design was chosen. The qualitative interview approach enabled an in-depth exploration of municipal employees’ experiences and perceptions concerning how they contribute to supporting children with diabetes and their families during institutional hours.

The study was approved by the Danish Data Protection Agency (P-2021-276) and carried out in accordance with the Declaration of Helsinki. According to Danish legislation, interview studies require no approval from an ethics committee.

### 2.1. Setting

Denmark is divided into five regions with a total of 98 municipalities.

In Denmark, institutions (schools, kindergartens and daycares) are required to ensure the support needed by children with conditions such as diabetes. Institutions are, however, not required to allocate a specific staff member with responsibility for the care needs of such children and adolescents [14]. It is the parents’ responsibility to ensure that institutional staff are provided with instructions about the child’s medication. Individual teachers and social workers are not required to conduct medical care. It is the principal’s responsibility to initiate procedures to ensure support for the child’s medical needs [15].

The institution can, in co-operation with the family and hospital, apply to the municipality for additional non-trained support staff in the form of a special needs assistant (SNA), who can help children with significant care needs. Parents can also apply for reimbursement for lost earnings and additional costs.

### 2.2. Participants and Recruitment

All 98 Danish municipalities were invited to participate in the interview study. The support of children with diabetes is not handled by a specific employment type or department and therefore, the municipalities were initially contacted by telephone to identify municipal employee(s) with knowledge about diabetes support for children in school, kindergarten, or daycare institutions. This recruitment process was particularly challenging due to difficulties identifying the key employee(s) in the first 35 contacted municipalities. Therefore, the remaining 63 municipalities were contacted via e-mail to the municipality’s main e-mail address. In the e-mail, the purpose of the study was briefly introduced, and the municipality was asked to appoint one or more relevant employee(s) with knowledge of the municipality’s support for families with a child with diabetes in school, kindergarten, or daycare institutions. In the absence of a response, a reminder was sent and, in the event of a continued lack of response, the municipalities were contacted by telephone.

The present study is based on 80 online interviews with 121 employees (Table 1) from 74 municipalities in Denmark; in some municipalities more than one employee participated. The interviews were conducted in April–September 2021.

### 2.3. Online Interviews

The interviews were semi-structured and included a range of open-ended questions designed to inform the overall objective focusing on differences, challenges, and potentials in diabetes support across municipalities. The interviews were primarily focused on the following three themes: (1) Framework and organizational structure, including communication, co-operations and departments involved in support of diabetes; (2) Experiences, focusing on actual case experiences; and (3) Future perspectives; areas in which they see potentials and challenges.

All interviews were conducted by either the first author or an additional research assistant using Microsoft Teams. This was convenient and easy for the municipal employees and enabling video allowed for a more smooth face-to-face dialogue. [16,17]. The interviews lasted 10–45 min.

### 2.4. Data Analysis

**Figure 1:**
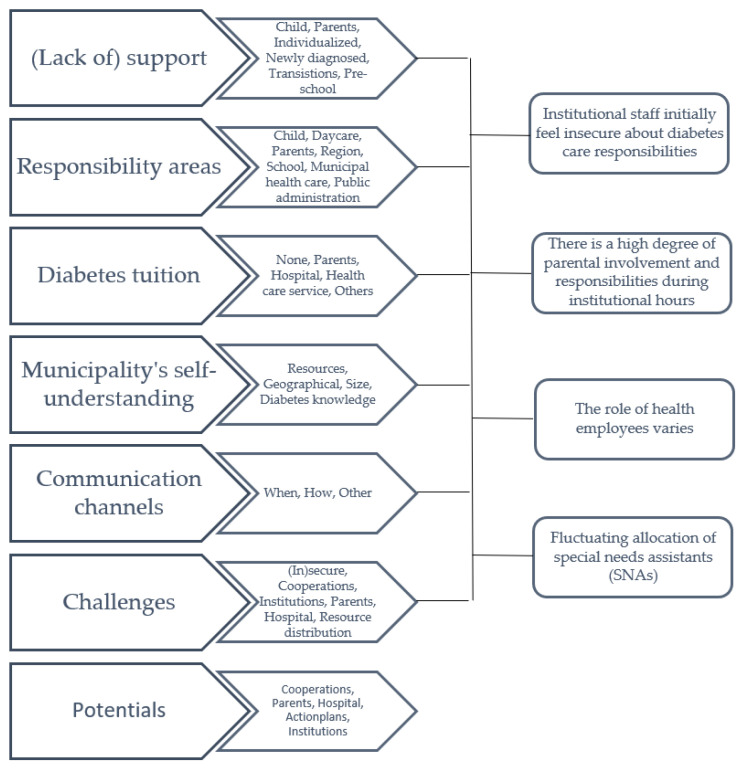
An overview of the code groups, subgroups and four main categories.

The recorded interviews were imported into QSR NVivo and analysed using qualitative content analysis [18]. The recordings were viewed, discussed, and compared by L.B.J. and D.G. In the coding process, data were organized into code groups that reflected the core topics relevant to the research question. The identified code groups were: (Lack of) support, responsibility areas, diabetes tuition, municipality’s self-understanding, communication channels, challenges, and potentials. Within each code group, we established subgroups, exemplifying different aspects of each code group and condensing the content of each meaning unit. Lastly, we synthesized all condensates from each code group and revised to form the four overarching main categories presented in the results. (Figure 1)

## 3. Results

### 3.1. Institutional Staff Initially Feel Insecure about Diabetes Care Responsibilities

Municipal staff members reported that the institutional staff felt insecure about diabetes management when a child with diabetes enrolled in the institution or returned after being diagnosed. The insecurity among the staff was described as one of the most significant challenges to diabetes care in institutions. The staff’s main concern and a source of great uncertainty was described as a fear of not knowing what to do in diabetes-specific emergencies. As a result of the insecurity, the institutional staff often refused to decide insulin doses, inject insulin, and do finger pricks. The staff experienced diabetes management tasks as being too far from their professional expertise:

“When I say that there is room for improvement, I mean we felt like incapable and alone. And thought: “is this really a school responsibility?”. Instead, shouldn’t the task be allocated to a school nurse or a nurse? It is a bit of a stretch for a schoolteacher to give a child insulin.” (Public administration employee/welfare secretary)

In relation to this, staff members felt overwhelmed when dealing with children with diabetes, which resulted in difficulties in assigning the responsibility for giving the necessary support to the children. In schools, the school principal has the responsibility for specific employee policies concerning how to support children with diabetes. The majority of the municipal staff members reported that the responsibility for diabetes management support was spread among several staff members and not specifically assigned to a contact person.

When a child had a specific contact person during school hours, it was not necessarily one of the child’s main schoolteachers. For example, it could be a janitor, a school nurse, a non-teaching support person or the school principal. These specific staff members were often chosen because they had diabetes themselves, had a family member with diabetes or had experience with diabetes from a former workplace. In some cases, the initial fear of diabetes management among the schoolteachers could also be the reason why another staff member was the first contact person for the child with diabetes:

“[…] And it’s a huge responsibility, they [schoolteachers] feel. So, it was the school principal who—because none of the schoolteachers would take the responsibility—had to be around the child. That’s not how it’s supposed to be.” (Municipal health service employee)

The greatest insecurity was experienced when none of the school staff members had prior knowledge of diabetes. This was reported as creating conflict regarding assigning the responsibility, and in some cases the schoolteachers’ union got involved and had formally advised their members not to be directly involved in diabetes care for children, as it is not a legal requirement. In these cases, meetings with different municipal staff members and the parents of the child with diabetes were held:

“[…] And this is where the discussion got really uncomfortable… That the school wouldn’t take the responsibility for measuring blood glucose and giving insulin to the child. The school felt insecure about the tasks, and the schoolteachers close to the child thought it was a great burden to teach a class and leave the classroom to measure blood glucose while 24 pupils are waiting and so on.” (Municipal health service employee)

Especially in these cases (but also in general), municipal staff members indicated that schoolteachers felt insecure about diabetes management and reported a strong need for a structured written plan for gaining knowledge and experience as their insecurity decreases with time.

“They [teachers] feel it’s a huge responsibility and maybe also that they don’t have enough time and knowledge to deal with high-risk diabetes situations. However, I think, when the children come and you get to know them and so on, then they handle it very well.” (Public administration employee/welfare secretary)

Municipal staff experienced that insecurity among institutional staff members created insufficient support in diabetes management in some institutions. These challenges were often experienced when no institutional staff members had any knowledge about diabetes management.

### 3.2. There Is a High Degree of Parental Involvement and Responsibilities during Institutional Hours

Resources within the family were seen as an important factor in determining how well-regulated the child’s diabetes was. For this reason, such resources are important for how demanding institutional staff find the tasks of supporting the child.

“[…] the schoolteachers faced huge challenges in terms of co-operation with parents. It’s my experience that when it’s a home with resources and they know how to support and provide the right conditions for their child… you know, not sending their child to school without breakfast and the children know how to react and ask for help etcetera. Then it does okay. […]” (Public administration employee/welfare secretary)

The extent of the families’ resources was reported to be an important factor in determining how demanding staff found the responsibility of supporting the child with diabetes. In relation to this, the families were described as an important resource for institutional staff members, as the parents were expected to be available to them at any time if they had any questions about diabetes management. For this reason, regular telephone contact between the institutional staff and the parents was normal.

“From my experience, it’s the parents who are at the centre of all of this. And it’s extremely important, because in this… they must have trust in us taking care of their child, and we need to have employees who can contact the parents. Always better one time too many. There’s reciprocity, so it’s okay to call on the phone if you have any doubts.” (Institutional employee)

Regular communication between institutional staff and parents was perceived as important. The communication included contact by telephone, visits to the school by parents, contact with staff at the beginning and/or the end of the day, or by keeping a logbook. Furthermore, the parents were perceived as mediators between the institution, the hospital, and other municipal staff members.

The role as communication link between different staff members included different and divergent expectations being placed on the parents by the various institutions, municipalities, and regions. Divergence in descriptions of the parents’ role was mostly seen in the institutional and municipal support:

“I’ve had cases where I had to tell the parents: “It’s hard to tell you this, but we don’t have the staff to do these tasks, so you’ll have to contact the social administration to get compensation for lost earnings, until we can get it under control”.” (Public administration employee/welfare secretary)

In contrast, other municipal staff members said:

“[…] it might often be them [schoolteachers and social workers] feeling that they don’t have the competences, they can’t take responsibility, and it’s too demanding… for this reason, they’ve got a feeling that we should cover loss of earnings and that’s not possible, because that’s only a possibility in the beginning.” (Public administration employee/welfare secretary)

The parents’ role as a communication link between the institution and the municipal staff was described not only as divergent because of the insecurity among institutional staff, but also as a fear among parents about their child not getting appropriate support during institutional hours.

“Well, it’s always challenging when parents demand more resources than they’ll get, as they have major worries about their child. I think we’ve had a single case in which the parents thought their child should be covered all the time. And we thought; no, it must happen in co-operation with the personnel who are already around the children.” (Public administration employee/welfare secretary)

The expected responsibilities of parents included providing information and guidance on diabetes care, either after the return of a child who was recently diagnosed or when a child with diabetes was enrolled in the institution. However, the overall diabetes training for the institutional staff varied greatly across institutions, municipalities, and regions. It was often the parents who provided all the training.

“Much of it [the responsibility] rests on the parents. On some level, I also think that they’re the right people. You know, the parents … they must be able to manage this, so I think it’s good that they know how to impart this knowledge.” (Municipal health service employee)

In contrast, other municipal staff reported that parents should not be expected to have sole responsibility for training institutional staff in supporting self-care management. For this reason, other municipal staff members meant that parents should be given the chance to ‘just be parents’, without having all responsibility during institutional hours.

Parental involvement affects the possibilities for creating sufficient support for children with diabetes during institutional hours. Large proportions of diabetes management support are dependent on resources within the family but finding the suitable balance for this involvement is difficult and too much or too little involvement creates frustrations for the school, the child and the parents.

### 3.3. The Role of Health Employees Varies

The role of municipal and regional health employees varied, but the municipal and regional nurses played a significant role in supporting institutional diabetes care. However, their role was not clearly defined. The regional nurses were generally perceived as having the overall formal role of teaching the institutional staff about diabetes physiology and management. The municipal nurses were perceived as having no specific role or responsibility in caring for and supporting children with diabetes and their families.

Most of the interviewed school nurses reported that, even though they had a background in nursing, they did not directly support diabetes management, because they did not have adequate knowledge:

“We aren’t the primary informants. We’re not supplying the information because we have a deep respect for this area. It’s so specialized and requires constantly updating diabetes knowledge.” (Municipal health service employee)

A few of the interviewed school nurses, especially those from smaller municipalities, were more involved in diabetes management, for example, by teaching and supporting schoolteachers or helping the child with insulin injections.

Most municipal health employees played no significant role in supporting children with diabetes and rarely received information when a child returned after being diagnosed or was enrolled in the institution.

“We ask for information about the children at school who get diabetes, and sometimes we get the information by coincidence during screening and we see these children: “oh, you have diabetes?”. We have no idea.” (Municipal health service employee)

The school nurses rarely got involved in supervising self-care, as their responsibility is in preventive healthcare. However, they could be a part of a supporting team because they often knew the families and had knowledge of the psychosocial aspects of having a chronic illness:

“It could be that they might experience difficulties in compliance. Typically, adolescents, I would say… don’t manage their diabetes, don’t take their insulin, measure blood glucose. How should we handle this and what are the school’s tasks really? And then it could be something like the teacher trying to draw attention to: “remember to measure your blood glucose”, but it might be considered a bit unfortunate, as it creates unwanted attention and that’s exactly what might be difficult for the child.” (Municipal health service employee)

The importance of supporting psychosocial difficulties was particularly highlighted when children are newly diagnosed or when they get older and want to fit in with a peer group, which often resulted in reduced blood glucose testing and insulin omission in institutional hours.

The communication, plans, and distribution of responsibilities between municipal and regional health employees varied a great deal. This municipal health employee described confusion regarding responsibility for an adolescent experiencing psychological challenges:

“[…] like psychological support when you stand out from peers and have to go to treatment and so on. I actually doubted how much we from the municipal side can support this group and how much the regional side takes care of.” (Municipal health service employee)

Some children with diabetes might ‘slip between the cracks’, as the definition of whether it was a municipal or regional responsibility was unclear. The degree of involvement differed across all institutions, municipalities, and regions, and there were no clearly defined responsibility areas or defined levels of involvement from regional and municipal healthcare professionals. This creates frustrations and hassle for everyone involved in institutional diabetes care.

### 3.4. Fluctuating Allocation of Special Needs Assistants (SNAs)

The jurisdiction to ensure diabetes management support in the institutions was described differently by the various municipal staff and affected the allocation of a special needs assistant (SNA), which was based on several different factors. The SNA is an additional non-teaching (and often non-trained) support person, who is not assessed automatically but for whom the school principal can apply for funding. One of the most significant factors for whether the institution will be granted an SNA was the legislative definition of diabetes as ‘a handicap’ or ‘an illness’.

When diabetes is defined as an illness, it is compared to, for example, having a bone fracture or epilepsy, which does not legally require provision of any institutional staff support:

“[…] legally, they have the opportunity to apply for what I call ‘single integration funds’. And that’s if the pupils are cognitively well-functioning, but may be in a wheelchair, blind, deaf or have other musculoskeletal disabilities. Then they can seek funding for that. But that’s not possible when it is diabetes, because we define that as a chronic illness that the pupil must learn to manage and live with. But that doesn’t mean there are fewer requirements for securing adequate support out there.” (Public administration employee/welfare secretary)

Other municipal staff described diabetes defined as ‘a handicap’ as diabetes that requires physical support and therefore, institutions in these municipalities can get funding for an SNA.

“As a diabetic child you shouldn’t have to compete with educational support. In this way we are trying to separate things and categorize diabetes as a disease that requires physical support.” (Public administration employee/welfare secretary)

Other factors for provision of diabetes-related support were likely to be individual, thus based on parameters such as diabetes duration, age, blood glucose regulation, family relations and other life conditions. Furthermore, the declaration from healthcare professionals was important:

“It’s based on what the treating part tells us… yes, whether it’s you or the hospital or whoever it might be, right? In most cases, if diabetes and medicine are regulated, then we don’t do more about it. But in the cases when it isn’t well regulated, and the child is at risk of shock or something… Well, then it’s this–depending on age, ability to perform diabetes self-care, and how familiar the child is with their own illness–that decides the assigned support.” (Public administration employee/welfare secretary)

The individual assessments of every single case were based on different parameters. Not knowing which parameters are essential for obtaining an SNA was described as a source of frustration for the institutional staff. For example, at one institution a social worker reported having two children with diabetes in the school. One child was young but described as too well-regulated, and the other child was too old to have an SNA assigned:

“We are applying for an SNA all the time. We have a child, who is very challenged with diabetes, but she is too old, so we can’t get an SNA for her.” (Public administration employee/welfare secretary)

There was significant frustration among the institutional staff members because crucial factors for allocation of an SNA were unspecific. The municipal staff members gave no specific age, duration or HbA1c levels for being assigned an SNA.

Allocation of an SNA was often based on the individual municipal staff member’s knowledge and/or view concerning how demanding diabetes care was during institutional hours:

“In light of what you said: “Do you get support?”. To me, it was just; wow, what do you need support for? You know, they aren’t disabled. They manage… with some precautions, right?” (Public administration employee/welfare secretary)

The legislative definition and institutional management of diabetes are often based on personal assessments, since in Denmark there is no clear jurisdiction concerning diabetes care in institutions. This generates a significant amount of confusion among families and institutional employees.

## 4. Discussion

This national qualitative study provided insights into the support provided or not provided for diabetes care of children enrolled in school, kindergarten, and daycare institutions in Denmark. We found four main themes that also constitute four significant deficiencies in the diabetes management support: (1) insufficient support in diabetes management across institutions, (2) a large proportion of diabetes management during institutional hours is dependent on parental involvement, (3) different levels of involvement from regional and municipal healthcare professionals, and (4) Danish legislation relevant to assignment of SNAs is interpreted differently across municipalities and institutions.

Our results support the notion that some teachers and social workers have concerns about experiencing ‘life-threatening’ episodes at institutional hours, which in our study was observed as an initial fear when a child with diabetes returns after diagnosis or enrols in the institution. We identified deficits in the support provided for adolescents with diabetes, either because they are expected to manage their diabetes on their own or because municipal employees had limited knowledge about who was responsible for the support adolescents needed. The lack of support for adolescents has been revealed in previous research [4].

Also in line with previous research, we found that the institutions are highly dependent on the parents. The fact that many parents cannot transfer the responsibility to institutional staff is important, as burn-out symptoms are common among parents of children with diabetes and are often associated with the feeling of diabetes affecting their everyday life [9,11,19]. In our study, the parents functioned as the communication link between institution, teachers/social workers, healthcare professionals and municipal employees. Similar findings were reported by Gutzweiler et al. [20], who stated that teachers experience poor communication between teachers, parents, and healthcare professionals.

Our study indicates that the role of school nurses in supporting children with diabetes is vague. They have responsibilities for preventive healthcare, but are rarely involved in supervising self-care in children with diabetes, as they are not informed when a child with diabetes enrols or returns after having been diagnosed. Instead, the child’s teacher, or another member of the school staff, has the responsibility for supporting the child in place of the parents. According to a literature review of the role of school nurses conducted by Stefanowicz and Stefanowicz [13], greater involvement on the part of school nurses could be effective and should have a positive effect on the condition, improvement of metabolic control, school activity and safety at school.

In general, the allocation of an SNA varied and depended on age, diabetes duration, and the declaration from regional healthcare professionals. The lack of consistent support for children with diabetes in schools is in line with previous studies in a Danish context [9] and in other Nordic countries [4,8].

Thus, the implications of the present study are the need for national regulations and legal clarification to ensure the distribution of responsibility and structured action plans for diabetes care in institutions as well as the need to reduce the burden on parents. Lastly, another possible improvement of self-care management in Danish school settings would be greater involvement on the part of the municipal nurse as a resource to provide support to institutional staff as well as efforts to improve communication between parents and the institution [21]. Moreover, nurses need to make sure that children and their institutional caretakers know the plan of action. This may relieve stress and anxiety among both parents and institutional staff members. However, municipal nurses are not always at the institutions, and research is needed to determine whether this support structure might provide better results for children and young people with diabetes and their families.

The methodology in our study was limited to an explorative qualitative investigation of municipal employees’ perceptions and meanings, as they were narrated in online interviews. However, we believe that the insights from the interviews are highly representative of the national support given to children with diabetes at institutions, as we interviewed 121 municipal employees from 74 out of the total of 98 Danish municipalities. The present study provided us with important results, but the municipal respondents’ area of responsibility varied greatly and was limited to their knowledge of children with diabetes who have difficulties and/or for whom requests for an SNA have been made.

More in-depth studies are needed to accurately describe the mechanisms revealed in our study. There is a need for macro-sociological studies of the overall structures as well as micro-sociological or anthropological studies of the relational aspects of everyday life in the institutions.

Moreover, potentials and challenges found in this study could be elaborated and developed into national regulations or guidelines. The effect of such national regulations needs to be closely monitored with a view to ensuring equal support for all children with diabetes.

## 5. Conclusions

In conclusion, the present nationwide qualitative study is the first to report on the support given to children and their families, as seen from a municipal perspective. We found that even though Denmark guarantees, by law, the child’s right to support in diabetes self-care in institutions, diabetes management in Danish institutions still needs to be improved to secure equal support for all children with diabetes.

## Figures and Tables

**Table 1:** Interviewed municipal employees.

Employment Category	Examples of Occupation	
Public administration employees/welfare secretary	Special educational consultant	
	School counsellor	61
	Psychological adviser	
Municipal health service employees	School nurse	37
	Municipal doctorHome nurse	
Institutional employees	School principal	23
	Social worker	
	Kindergarten manager	
